# Soil Fungal Diversity Loss and Appearance of Specific Fungal Pathogenic Communities Associated With the Consecutive Replant Problem (CRP) in Lily

**DOI:** 10.3389/fmicb.2020.01649

**Published:** 2020-07-15

**Authors:** Guiying Shi, Hongqiang Sun, Alejandro Calderón-Urrea, Xixia Jia, Hongyu Yang, Guoli Su

**Affiliations:** ^1^College of Horticulture, Gansu Agricultural University, Lanzhou, China; ^2^College of Plant Protection, Gansu Agricultural University, Lanzhou, China; ^3^Department of Biology, College of Science and Mathematics, California State University, Fresno, Fresno, CA, United States

**Keywords:** food lily, monoculture, soil health, consecutive replant problem, fungi, metabarcoding analysis, diversity

## Abstract

Edible lily (*Lilium davidii* var. *unicolor*) has economic value in China, particularly in Gansu Province, due to its uses as food and in gardening. Edible lily is usually cultivated in a long-term continuous monoculture resulting in the so-called consecutive replant problem (CRP), which is associated with severe soil degradation and significant yield and quality losses. This study was conducted to investigate the fungal community structure and specific fungal members related to lily’s CRPs using metabarcoding analysis. Fungal diversity of rhizosphere soil was analyzed by high-throughput DNA sequencing (Miseq) of samples collected in fields at 0, 3, 6, and 9 replant years (L0, L3, L6, and L9, respectively). The results show that long-term replanting significantly decreased both soil fungal diversity and abundance at the OTUs levels. Furthermore, replanting altered the soil microbial communities, where 4 to 5 years of replanting is a key transition period for substantial change of fungal community structure, resulting in new fungal community structures in L6 and L9 compared to in L0 and L3. The fungal diversity loss and fungal community structure simplification contributes to the negative effect of replanting in lily, and after 6 years of replanting, accumulation of highly abundant pathogenic fungal genera and depletion of the putative plant-beneficial fungal genera exacerbate the lily CRP. In addition, changes in the soil physiochemical properties strongly contributes to the new structure of fungal communities, and the genera *Cryptococcus* and *Guehomyces* could be regarded as potential indicators to monitor and manage sustainable soil health in the lily cropping system.

## Introduction

The Lanzhou lily (*Lilium davidii* var. *unicolor*) is the only edible sweet lily consumed as food in China. The lily is an endemic species with a narrow distribution that is only suitable to the arid region, at the altitude of 2000–2600 m, in Gansu Province, western China. The lily is propagated asexually and cultivated perennially, so it is usually cultivated in a long-term continuous monoculture, which results in serious consecutive replant problems (CRPs), such as severe soil degradation and significant yield and quality losses. In addition, the CRP in lily causes an estimated 50 million dollars lost for farmers in this area. To address CRPs, local famers usually apply high levels of organic fertilizer every year to maintain a sustainably quality of soil; however, the CRP still becomes increasingly severe with each replant year, and after six years of replanting, the lily CRP is so severe that the farmers usually rotate the field with other crops or leave it idle. Therefore, a better understanding of the possible causes of the lily CRP will help monitor and manage sustainable soil health in the lily cropping system.

Changes in a soil microbial community is regarded as one of the main factors of CRPs (Larkin, 2008). Many reports have revealed the negative effects of the soil microbes on plant growth and yield, and discussed some important bacterial and fungal communities related to CRPs (Franke-Whittle et al., 2015; Xiong et al., 2015; She et al., 2017). For instance, compared to the vanilla monoculture system, in a black pepper-vanilla mixed system, the lower abundance of *Fusarium oxysporum* in rhizosphere soil and the increase in putative beneficial plant fungal genera explained the healthy growth of vanilla in the soil of a long-term continuously cropped black pepper field (Xiong et al., 2016). Accumulations of fungal pathogens at the expense of beneficial plant fungi were also observed in consecutively cropped peanut soil (Li et al., 2014).

Soil fungi diversity has been investigated in crop monoculture systems. Some researchers believe that increased fungal diversity may be a common phenomenon in monoculture agroecosystems (Chen et al., 2012; Liu et al., 2014; Xiong et al., 2015), while some reports have revealed different results such as in the apple replant system, where fungal diversity is stable during the replant years (Franke-Whittle et al., 2015) and in the black pepper-vanilla system, where fungal diversity is significantly higher than that in the vanilla alone monoculture system (Xiong et al., 2016). In nature, soil biodiversity has a positive correlation with the productivity and sustainability of a system (Hunt and Wall, 2002), and loss and simplification of soil community composition impair multiple ecosystem functions, including plant diversity, decomposition, nutrient retention, and nutrient cycling (Wagg et al., 2014). Soil biodiversity is sensitive to changes in land management practices, such as cropping systems, tillage and fertilization (Roy et al., 2018).

The possible causes of CRP might be an interaction involving soil biotic and potentially abiotic factors (Kanfra et al., 2018; Winkelmann et al., 2019). In the lily replanting system, within one 3-year cycle, the above-ground plant residue is usually left in the field every winter for 2 years, and the bulbs and plants are harvested during the third year. It has been shown that crop residue retention appears to increase soil microbial biomass (Salinas-Garcia et al., 2001), so we speculated that this practice in lily enhances disease occurrence, especially in terms of soil-borne diseases. Our team has identified several pathogenic *Fusarium* spp., which resulted in Lanzhou lily wilt disease from the lily replanting soil where this disease occurred (Bian et al., 2016). Thus, we hypothesize that accumulation *Fusarium* spp. might play an important role in the lily CRP occurrence. Moreover, other fungal members, which belong to the well-known root-rot complex, may be important contributors to the CRPs, such as *Rhizoctonia solani, Phytophthora* spp., *Cylindrocarpon* spp., and *Pythium* spp. (Mazzola, 1998; Manici et al., 2003; Manici and Caputo, 2010; Kelderer et al., 2012). However, since different biological agents have been implicated in disease development, there is no typical symptom occurrence with CRPs, so it is difficult to diagnose a CRP by only aboveground symptoms. A recent publication reported soil fungal community dynamics of Lanzhou lily in 3 years of one cultivation cycle (Hua et al., 2019), however, their dynamics under long term replant system and their roles in the lily CRPs have not been reported.

In this study, we collected data on soil fungal communities based on high-throughput DNA sequencing techniques (Miseq) to investigate the replant-year-related dynamics of the fungal composition and structure in the lily rhizosphere from fields with continuous cropping histories for 0, 3, 6, and 9 years. The goal of this project was to determine if replanting affects fungal community structure and diversity, which has been demonstrated in other crops, and if this is the case, what are the specific groups of microorganisms that are detected. Similarly, we wanted to explore the specific fungal members related to the cause of the lily CRP.

## Materials and Methods

### Field Description and Sampling

The sample site was located in Jiangjiashan village, Lintao County, Gansu Province (western China, 103°53′12′′∼ 103°53′14′′E, 35°49′11′′∼ 35°49′13′′N, 2330 m elevation). The soil is locally known as Huangmian soil (a deep soil layer, high water–storage capacity, pH 7.8, and organic matter 1.3%). Lanzhou lily has been cropped as food in this area for over 140 years.

Because Lanzhou lily typical grows 3-year cycles before harvest, we designed 4 treatments: L0 (non-replant lily), L3 (crop lily for 3 years), L6 (monocrop lily for 6 years), and L9 (monocrop lily for 9 years). These treatments were collected from four fields that were managed agronomically in a similar manner, with similar fertilization regime in their cropping history, as well as if they were previously planted crops. We identified 20 sampling sites within these four fields for sample collection: we collected five samples from each of the four fields. One soil sample in L3 was excluded from analysis because only 1890 reads were obtained from the high-throughput sequencing; so only four samples were left in L3. In each sampling site, a 66 m^2^ (11 m × 6 m) size plot was chosen. The L0 field had previously been planted with lily and was idle for the previous year. The L3 field had been planted with lily for the last 3 years and the food bulbs were harvested in the previous autumn. The L6, L9 fields had been planted with lily for the last 6, 9 years and the food bulbs were harvested two and three times, respectively. The lily cultivar was sowed with density of 0.30 m × 0.15 m per plantlet in March 28, 2016 (the bulb seed was approximately 17 ± 2 g), and managed with the same agronomic management and fertilization regime based on traditional agricultural practices in this region with an organic fertilizer (containing sheep or chicken manure 30 m^3^/ha each year) and without irrigation.

During August to September, it often rains in this area, and if it rains too long, the lily might get *Botrytis cinerea* and die soon. To avoid the disease, soil sampling was conducted at the lily flowering stage on July 18, 2016. Each plot was subdivided in four subplots of the same size, and five plants were randomly selected within each subplot, thus a total of 20 plants were collected in each plot. The rhizosphere soil samples were obtained from the soil adhering to plants roots (plants were gently shaken by hand), and the rhizosphere soils from the 20 plants were mixed to generate one soil sample; all the 20 soil samples were collected in this same manner. Each soil sample was divided into two subsamples: one was brought to the laboratory on dry ice and stored at −80°C for downstream applications (DNA extraction). The remainder sample was air-dried for soil characteristic analysis.

### Determination of Soil Physiochemical Properties

Soil pH was measured using an HI8314 portable pH meter (Hanna Instruments, Italy). The pH was measure by mixing 1 volume of soil with 1.5 volumes of distilled water and taking a reading of the mix. Bulk density was measured by the cutting ring method. Salt content was measured by dregs drying-Quality method. Moisture content was measured by oven-drying method. Organic matter was measured by potassium dichromate method, available nitrogen was measured by alkali-diffusion method, available phosphorus was measured by Mo-Sb colorimetric method, and available potassium was measured by flame photometry method. These eight methods were conducted as previously described (Liu et al., 2009).

### DNA Extraction

Soil DNA was extracted using a PowerSoil DNA Isolation Kit (MoBio Laboratories, Carlsbad, CA, United States) following the manufacturer’s instructions. The purity and quality of the genomic DNA samples were checked by 0.8% agarose gels electrophoresis, and the concentration was measured using a NanoDrop ND-2000 spectrophotometer (NanoDrop Technologies, Wilmington, DE, United States).

### PCR Amplification and High-Throughput Sequencing by Illumina Miseq

The ITS1 region was chosen because it is highly variable and optimal for the shorter reads available with the paired-end Illumina MiSeq. The amplicons were generated with the forward primer ITS1-F (5′-CTTGGTCATTTAGAGGAAGTAA-3′) and the reverse primer ITS1-R (5′-TGCGTTCTTCATCGATGC-3′) (Reazin et al., 2016). For each soil sample, a 10-digit barcode sequence was added to the 5′ end of the forward and reverse primers (provided by Allwegene Company, Beijing). PCR was carried out on a Mastercycler Gradient (Eppendorf, Germany) using 25 μL reaction volumes containing 12.5 μL 2 × Taq PCR MasterMix, 3 μL BSA (2 ng/μL), the 2 Primers (5 μM each), 2 μL template DNA, and 5.5 μL dd H_2_O. The cycling parameters were 95°C for 5 min, followed by 32 cycles of 95°C for 45 s, 55°C for 50 s and 72°C for 45 s with a final extension at 72°C for 10 min. Three PCR products per sample were pooled to mitigate reaction-level PCR biases. The PCR products were purified using a QIAquick Gel Extraction Kit (QIAGEN, Germany), and quantified using Real Time PCR. Deep sequencing was performed on the Illumina Miseq platform at Allwegene Company (Beijing, China).

### Data Analysis

The raw data was first screened, and sequences were removed from consideration if they were shorter than 130 bp, had a low quality score (≤20), contained ambiguous bases or did not exactly match primer sequences and barcode tags. The software package Usearch (version 8.1) [Search and clustering orders of magnitude faster than BLAST] was then used to further filter out sequences which were erroneous or chimeric. The remaining high-quality sequences were clustered into operational taxonomic units (OTUs) at a 97% similarity level using UPARSE based on *de novo* genome assembly. Low-abundance OTUs (fewer than 2 reads, including singletons), which might influence richness and diversity estimates, were excluded from the subsequent analyses. Rarefaction (Amato et al., 2013), Shannon-Wiener (Maughan et al., 2012), Rank-Abundance (Bates et al., 2013), and alpha diversity index with Chao1, Good’s coverage, observed species, PD whole tree and Shannon index (Schloss et al., 2011) were calculated using the software Mothur (Schloss et al., 2009). The taxonomy of ITS sequences were analyzed against the UNITE database^1^ and the NT database^2^.

To examine the statistical significance of structural similarity among communities across sampling locations, both Principal Component Analysis (PCA) applying Euclidean distance metrics (Wang et al., 2012) and hierarchical clustering based on the UPGMA distance matrix (Sun et al., 2014) were performed using the R package. Analysis of similarities (ANOSIM) was calculated to compare the intra- and inter-group similarities based on the UniFrac distance (Noval Rivas et al., 2013). To compare community characteristics in greater detail, heat maps at genus level were constructed and Venn diagrams at OTUs level were created with the R package.

To determine the significantly important microbial taxa, the Linear Discriminant Analysis Effect Size (LEfSe) analysis was carried out as previously reported (Segata et al., 2011). To reveal the interaction within the fungal communities, network analysis was performed among the top 30 genera across L6 and L9 treatments. Correlations between genera were generated using a Spearman correlation analysis, *P* < 0.05 and |R| > 0.6 were used to construct the networks using Cytoscape (version 3.3.0). To further assess the relationship between the fungal community composition and physicochemical properties in different soil treatments, a redundancy analysis (RDA) was performed based on fungal abundance at OTUs level and physicochemical parameters using the R package.

### Accession Numbers

The sequences obtained in this study have been submitted to the NCBI Sequence Read Archive (SRA) under the Bioproject ID PRJNA550018.

## Results

### Quality of Fungal Diversity in the Replant Soil

Sequencing of the ITS1 region of the nuclear ribosomal DNA resulted in a total of 799224 high quality, chimera-free reads accounting for 266357120 bp with a dominant length of 200–260 bp. Among the reads, 227813, 169034, 196948, and 205429 sequences were obtained for each of the L0, L3, L6, and L9 soil samples, respectively. To normalize read depth, all samples were randomly sub-sampled to 27050 reads/sample.

**FIGURE 1:**
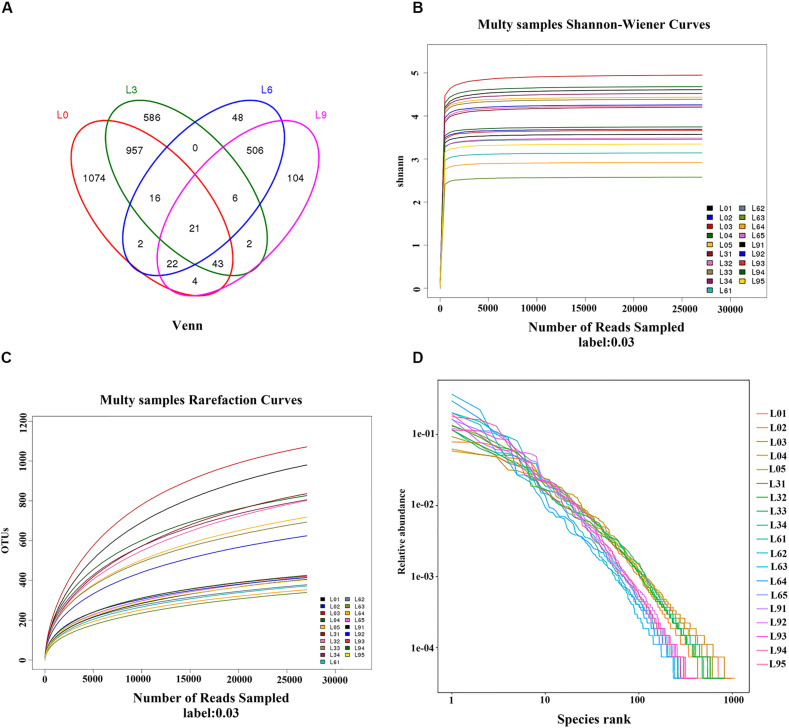
Venn diagram of unique and shared OTUs **(A)** and rarefaction curves **(B)** and Shannon-Wiener curves **(C)** and Rank-Abundance **(D)** of Lanzhou lily replant soil fungal communities from Illumina Miseq data.

Fungal diversity was measured based on OTUs. The OTU distribution Venn analysis demonstrated that there were a total of 3475 OTUs, and 1074, 586, 48, and 104 unique OTUs in L0, L3, L6, and L9, respectively (Figure 1A). The rarefaction analysis (Figure 1B) indicated that OTUs were complete in all of the generated libraries. Both the Shannon-Wiener analysis (Figure 1C) and Good’s coverage analysis (the Good’s coverage value between 98.91%∼99.57% were used in this analysis) also indicated that the sequence amount was enough to represent the true microbes in the sample, and deeper sequencing identification was successful. In addition, the Rank-Abundance analysis revealed the OTU evenness and abundance depleted among L0, L3, and L6, and then enriched in L9 (Figure 1D).

### Overall Structural Changes in Fungal Communities in the Replant Soil

**FIGURE 2:**
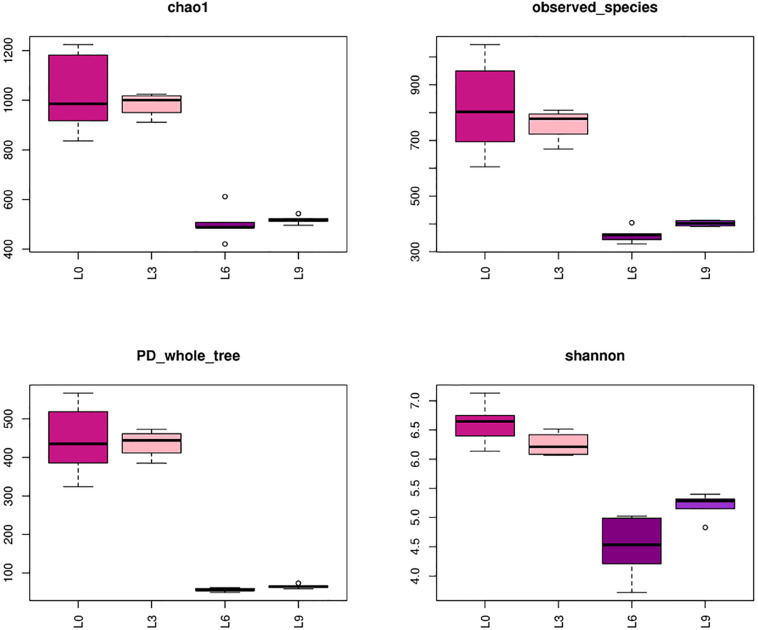
Fungal alpha diversity indices (at OTU similarity) of Lanzhou lily replant soil from Illumina Miseq data.

Alpha diversity indices were evaluated based on OTUs. Chao1 richness, observed species, PD whole tree and Shannon’s diversity revealed depletion of OTU similarity in L6 and L9 compared to L3 and L0 (Figure 2).

**FIGURE 3:**
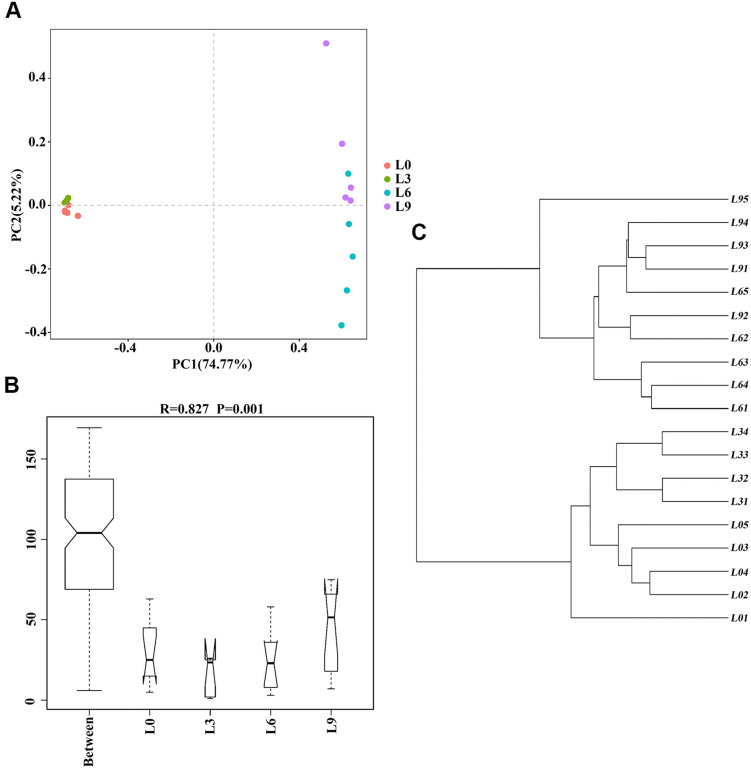
Overall structural changes in Lanzhou lily replant soil from Illumina Miseq data: PCA applying Euclidean distances distance metrics **(A)**; hierarchical clustering of soil fungal communities by UPGMA **(B)**; ANOSIM for group division [*P*-value between two treatments was L9-L0 (*P* = 0.011), L3-L0 (*P* = 0.016), L3-L9 (*P* = 0.008), L6-L0 (*P* = 0.01), L6-L9 (*P* = 0.031), and L6-L3 (*P* = 0.009), respectively] **(C)**.

The PCA based on OTUs was partially successful in representing sample data. The results showed that the samples grouped according to the soil sample replant years (Figure 3A). The replicates of each sample were found to group together, and all 4 groups for fungi differed, indicating a significant shift in rhizosphere fungal structure over lily replant years. Moreover, these 4 groups were clearly separated into 2 clusters: the L0 and L3 fungal community and the L6 and L9 fungal community. The first principle component axis (PC1), which contributed 75.44% of the total variation, and the second component axis (PC2), which contributed 5.22% of the variation, explained 79.99% of the variation. The pattern was confirmed by hierarchical clustering, which showed that the replicates of L0 and L3 were separated and that the replicates of L6 and L9 were not clearly separated (Figure 3B). Furthermore, the ANOSIM comparison between treatments revealed that the four groups differed significantly (*P* < 0.05) (Figure 3C). The results suggested that different years of consecutive monoculture lead to changes in fungal community, and especially by 6 years of replanting, the overall fungal community changed substantially.

### Taxonomic Distributions of Fungi Accumulated in the Replant Soil

**FIGURE 4:**
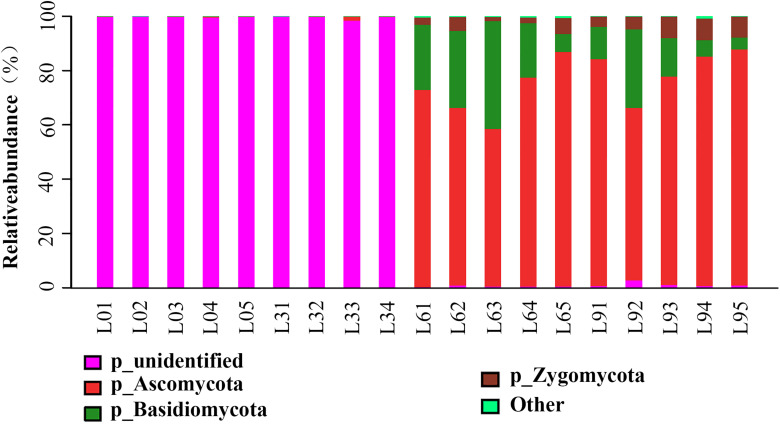
Barplots showing the distribution of all phyla present in Lanzhou lily replant soil.

**FIGURE 5:**
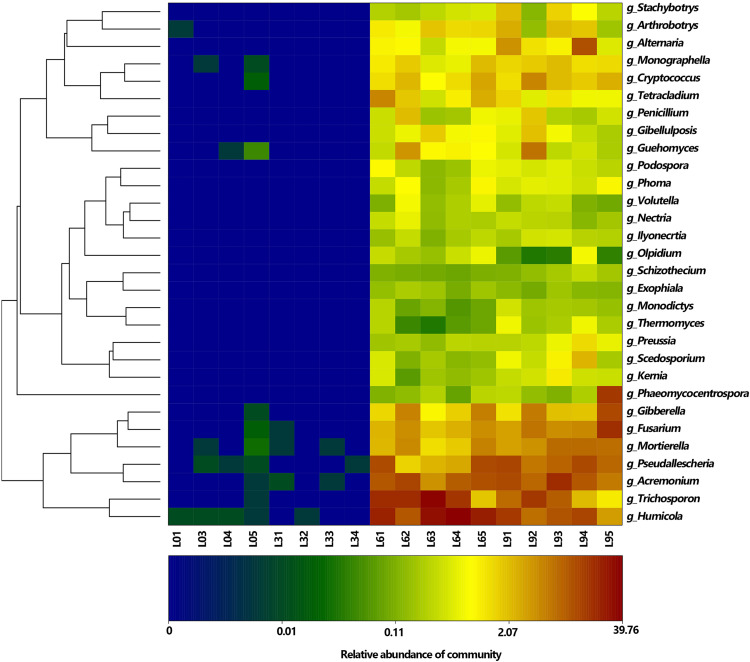
Heat map showing the distribution of top 30 genera (The relative abundance for the fungal genera are depicted by the color intensity, and the color key is at the bottom).

At all taxonomic levels in L0 and L3, a large number of OTUs could not be identified based on UNITE database. After 6 years of replanting, however, most of the unidentified groups disappeared, and many identified groups appeared at all taxonomic levels (Figures 4, 5). There were five phyla in which RA rapidly increased with replant years and accounted for 0.19, 0.49, 99.00, and 98.76% of the total fungi in L0, L3, L6, and L9, respectively. The most abundant phyla were *Ascomycota*, *Basidiomycota*, and *Zygomycota* (average RA > 2%), which accounted for 0.19, 0.49, 99.07, 98.55% in L0, L3, L6, and L9, respectively. Among them, *Ascomycota* accounted for 0.16, 0.49, 71.90, and 79.11% in L0, L3, L6, and L9, respectively. However, there were 0.57 and 1.24% sequences that remained unclassified in L6 and L9, which indicated that the soil contained few unknown fungal members. Now, sixteen classes were identified; the most abundant classes were *Sordariomycetes*, *Tremellomycetes*, and *Dothideomycetes* (average RA > 2%), which accounted for 0.03, 0.01, 86.95, and 82.87% of the total fungi in L0, L3, L6, and L9, respectively, and among them, *Sordariomycetes* accounted for 0.02, 0.01, 61.97, and 61.58% in L0, L3, L6, and L9, respectively. However, there were 1.41 and 2.30% sequences that remained unclassified in L6 and L9, respectively. The most abundant orders were *Hypocreales*, *Sordariales*, *Microascales*, *Trichosporonales*, and *Mortierellales* (average RA > 2%), which accounted for 0.02, 0.01, 83.89, and 81.15% of total fungi in L0, L3, L6, and L9, respectively. Among them, *Hypocreales* accounted for 0.01, 0.01, 22.90, and 31.23% in L0, L3, L6, and L9, respectively.

At genera level, 136 genera were detected, and among them, the most abundant genera were *Humicola*, *Trichosporon*, *Acremonium*, *Pseudallescheria*, *Fusarium*, *Mortierella* (average RA > 2%), which accounted for 0.01, 0.01, 67.53, 52.68% of the total fungi in L0, L3, L6, and L9, respectively (Figure 5). At the specie level, 146 species were detected, and among them, the most accumulated species (average RA > 2%) were *Humicola nigrescens*, *Acremonium nepalense* and *Pseudallescheria fimeti*. In addition, 3 *Fusarium* species were identified, which accounted for 5.98% of the total genus *Fusarium* sequences.

**FIGURE 6:**
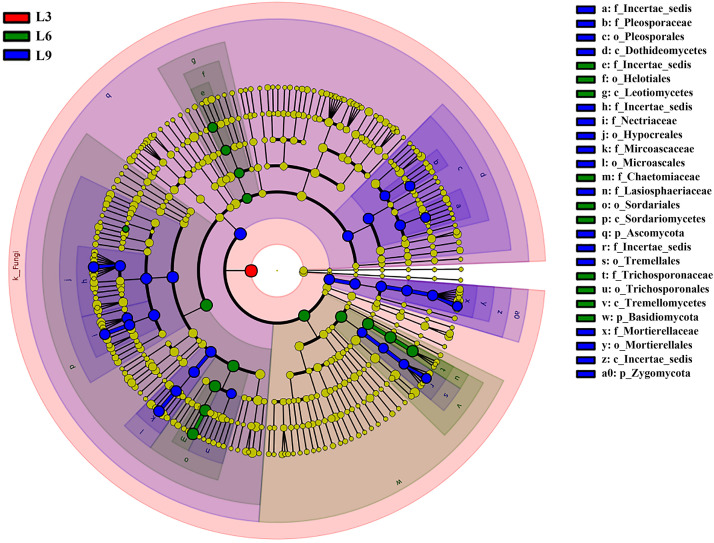
Cladogram obtained from LEfSe analysis showing changes in relative abundance of fungal communities at different taxonomic levels among different replanting years of Lanzhou lily from Illumina Miseq.

Cladograms obtained from the LEfSe analysis provided a deep insight into the changes of identified fungi accumulated in the rhizosphere soil at different taxonomic levels among different replanting years (Figure 6). Firstly, no dominant group was identified at the L0 and L3 treatments (Figures 4, 5). On the other hand, major dominant groups were identified in L6 and L9 treatments. Notably, the rhizosphere of the lily in L6 and L9 treatments were preferentially colonized by the phyla *Ascomycota* and *Basidiomycota*, and each phylum was represented by several dominant subsets of fungal groups. These dominant fungal group changes were as follow: (i) In dominant class *Sordariomycetes* of phylum *Ascomycota*, the most abundant orders *Sordariales* and *Helotiales* in L6 changed into *Microascales*;, moreover, there were other two orders *Pleosporales in* class *Dothideomycetes* and *Pleosporales in* class *Dothideomycetes*, which were dominant in L9; (ii) In dominant class *Tremellomycetes* of phylum *Basidiomycota*, the most abundant order *Trichosporonales* in L6 changed into *Tremellales* in L9; and (iii) Finally, the phylum *Zygomycota*, the order *Mortierellales* was dominant in L9.

### Replant Year-Related Dynamics of the Fungal Community Structure

The fungal community structures of short-term replant soil were significantly different from those of long-term replant soil. At the phylum, class and order levels, all of the dominant members, which appeared after 6 years of replanting, accumulated with replant year (Figures 4, 6).

Among all the identified genera, 91 genera were significantly shifted with replant years (*P* < 0.05). We list 27 genera (average RA > 0.1%) (Table 1), in which only 11 genera were detected in L0 and L3 with low RAs, and 16 genera were too rare to be detected or might not have existed in L0 and L3. Among the dominant genera (average RA > 1%), compared to in L6, the genera *Humicola* and *Trichosporon* were depleted in L9, while the genera *Acremonium*, *Pseudallescheria*, *Fusarium*, *Mortierella*, *Gibberella, Alternaria*, and *Cryptococcus* accumulated in L9.

**TABLE 1:** Genera that significantly changed (*P* < 0.05) with average relative abundance > 0.1% of total fungal reads in Lanzhou lily replant soil from Illumina Miseq data: Only OTUs with average relative abundance > 0.5% were noted.

Replant year	L0	L3	L6	L9
**(a) Depleted genera in L9 compared to L6 replant years**				
*g__Humicola*(OTU-3, OTU-5)	0.01%	0.00%	25.60%	10.32%
*g__Trichosporon*(OTU-1)	0.00%	0.00%	19.19%	7.47%
*g__Tetracladium*(OTU-23)	0.00%	0.00%	2.26%	0.94%
*g__Gibellulopsis*	0.00%	0.00%	0.89%	0.79%
*g__Penicillium*	0.00%	0.00%	0.73%	0.67%
*g__Nectria*	0.00%	0.00%	0.32%	0.25%
*g__Volutella*	0.00%	0.00%	0.36%	0.21%
*g__Olpidium*	0.00%	0.00%	0.35%	0.18%
**(b) Accumulated genera in L9 compared to L6 replant years**				
*g__Acremonium* (OTU-2)	0.00%	0.00%	9.93%	11.32%
*g__Pseudallescheria* (OTU-7, OTU-1773)	0.00%	0.00%	6.37%	10.02%
*g__Fusarium*(OTU-4)	0.00%	0.00%	3.26%	7.60%
*g__Mortierella*(OTU-16)	0.01%	0.00%	3.18%	5.94%
*g__Gibberella*(OTU-6)	0.00%	0.00%	3.06%	4.90%
g__*Alternaria* (OTU-12	0.00%	0.00%	0.73%	3.74%
*g__Cryptococcus*(OTU-10)	0.00%	0.00%	1.83%	2.64%
*g__Phaeomycocentrospora*(OTU-11)	0.00%	0.00%	0.19%	3.38%
*g__Guehomyces*(OTU-25)	0.01%	0.00%	1.40%	1.65%
*g__Monographella*(OTU-46)	0.00%	0.00%	1.25%	1.68%
*g__Arthrobotrys*	0.00%	0.00%	1.36%	1.46%
*g__Stachybotrys*	0.00%	0.00%	0.34%	1.02%
*g__Scedosporium*	0.00%	0.00%	0.23%	0.97%
*g__Phoma*	0.00%	0.00%	0.50%	0.57%
*g__Preussia*	0.00%	0.00%	0.23%	0.65%
*g__Podospora*	0.00%	0.00%	0.43%	0.44%
*g__Kernia*	0.00%	0.00%	0.22%	0.53%
*g__Ilyonectria*	0.00%	0.00%	0.24%	0.33%
*g__Thermomyces*	0.00%	0.00%	0.10%	0.40%

*Only OTUs with average relative abundance > 0.5% were noted.*

At the OTU level, 3391 OTUs were detected, and among them, there were 39 dominant OTUs (average RA > 0.5%), which accounted for 48.16, 53.90, 81.41, and 73.05% of the total sequence in L0, L3, L6, and L9, respectively. Moreover, all these 39 dominant OTUs (average RA > 0.5%) significantly shifted with replant years (*P* < 0.05), including 24 OTUs unidentified groups. After 6 years of replanting, 20 OTUs disappeared, and 19 new OTUs appeared. The significant change in the dominant depleted genus *Humicola* proportion was mainly driven by the depleted shifts in OTU-5 and OTU-3, and the proportion of the depleted genus *Trichosporon* was mainly driven by the depletion of OTU-1. For the accumulated dominant genera *Acremonium*, *Pseudallescheria*, *Fusarium*, *Mortierella, Gibberella*, *Alternaria*, and *Cryptococcus*, the proportions were mainly driven by the significantly enhanced shifts in OTU-2, OTU-7 and OTU-1773, OTU-4, OTU-16, OTU-6, OTU-12, and OTU-10, respectively (Table 1).

### Network Analysis

**FIGURE 7:**
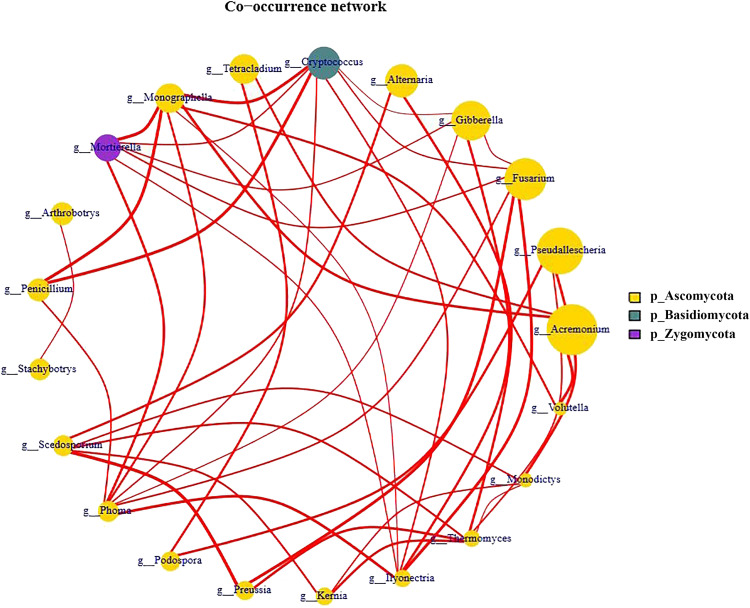
Network analysis of the fungal genera associated with L6 and L9 treatments of Lanzhou lily from Illumina Miseq.

To reveal the interaction within the fugal communities across the lily replanting processes, we constructed a co-occurrence network at the genus level in the replant system (Figure 7). The genera *Phoma*, *Monographella*, and *Cryptococcus* were the most densely connected nodes (degree = 7) and acted as “hubs” (highly connected genera). The genus *Phoma* co-occurred with the genera *Penicillium*, *Mortierella*, *Monographella*, *Cryptococcus*, *Gibberella*, *Fusarium*, and *Ilyonectria*. The genus *Monographella* co-occurred with *Mortierella*, *Phoma*, *Ilyonectria*, *Acremonium*, *Volutella*, *Cryptococcus*, and *Monographella*. Finally the genus *Cryptococcus* co-occurred with *Monographella*, *Mortierella*, *Penicillium*, *Phoma*, *Ilyonectria*, *Fusarium*, and *Gibberella*. In addition, the genera *Fusarium* and *Ilyonectria* were the second more important key groups (degree = 6).

### Links Between Fungal Community and Soils Physicochemical Properties

Soil physicochemical properties changed with the lily replant years (Table 2). Soil pH, salt content, organic matter, available nitrogen, available phosphorus were increased significantly (*P* < 0.05) with replant years. However, bulk density, water content, and available potassium decreased significantly.

**TABLE 2:** Summary of physicochemical properties of Lanzhou lily replant soil.

Treatments	Bulk	Moisture	pH	Salt	Organic	Available	Available	Available
	density	content		content	matter	nitrogen	phosphorus	potassium
	(g⋅cm^–3^)	(%)		(g⋅kg^–1^)	(g⋅kg^–1^)	(mg⋅kg^–1^)	(mg⋅kg^–1^)	(mg⋅kg^–1^)
L0	1.33 ± 0.01^a^	14.63 ± 0.09^a^	7.76 ± 0.03^c^	0.48 ± 0.01^d^	13.21 ± 0.10^d^	65.16 ± 1.01^c^	94.48 ± 0.93^d^	236.55 ± 2.8^a^
L3	1.32 ± 0.01^ab^	14.20 ± 0.21^b^	7.87 ± 0.03^b^	0.58 ± 0.01^c^	13.95 ± 0.13^c^	71.29 ± 0.86^b^	146.18 ± 5.98^c^	219.71 ± 3.54^b^
L6	1.31 ± 0.01^b^	13.19 ± 0.11^c^	7.90 ± 0.03^ab^	0.80 ± 0.01^b^	14.74 ± 0.13^b^	82.57 ± 0.99^a^	194.33 ± 2.59^b^	204.89 ± 3.69^c^
L9	1.30 ± 0.01^b^	12.09 ± 0.07^d^	7.99 ± 0.03^a^	1.08 ± 0.04^a^	16.18 ± 0.38^a^	83.25 ± 0.80^a^	209.95 ± 3.35^a^	171.04 ± 3.25^d^

*Different letters, within each column (marked as “a”, “ab”, “b”, “c”, and “d” in descendent value), represent the values of the statistical results at p < 0.05.*

**FIGURE 8:**
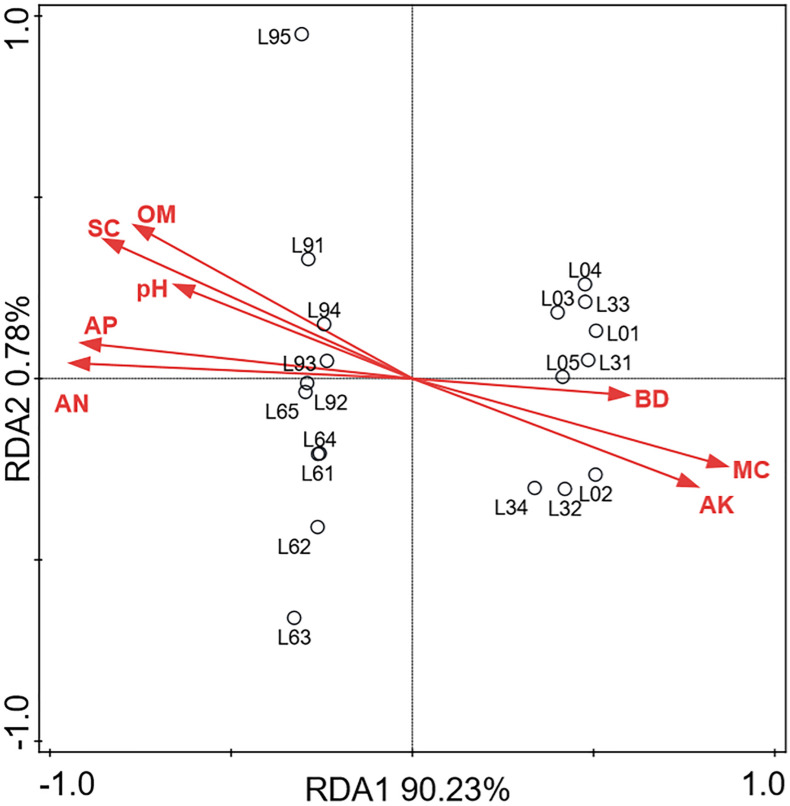
Redundancy analysis (RDA) of fungal communities (OUT) and soils physicochemical properties (Each sample is represented by black circles. BD, SC, MC, OM, AN, AP, AK represented bulk density, salt content, moisture content, organic matter, available nitrogen, available phosphorus and available potassium, respectively).

The RDA model was applied to investigate the relationships among these soil parameters and the fungal community compositions (Figure 8). Associations between the fungal groups and soils physicochemical properties were evaluated using the soil parameters data as the explanatory matrix. RDA1 and RDA2 explained 91.01% of the total variance, and all the soil physicochemical properties clearly correlates with the fungal groups identified. The RDA plot of the fungal community composition also clearly showed that the samples in L0 and L3 significantly differed with the samples in L6 and L9. The RDA model also indicated that salt content, available nitrogen, organic matter, available phosphorus and pH play an important role in shaping fungal community structure in L9 samples, and pH was closely correlated to the salt content.

## Discussion

### Fungal Abundance and Diversity in the Replant System

Both hierarchical clustering and PCA analyses indicated that changes in the fungal community composition were strongly linked to the replant years (Figures 3A,B). Alpha diversity indices (Figure 2) and RDA plot (Figure 8) clearly showed that the fungal community composition in L0 and L3 significantly differed with that in L6 and L9 samples. These indicate that 4–5 years of replanting is a key transition period for fungal community structure substantial change, that result in shaping completely the new fungal community structures in L6 and L9.

The results of our work also showed that long-term replanting significantly decrease fungal alpha diversity at OTUs levels (Figure 2). which are not in agreement with the conclusion from research on peanuts (Chen et al., 2012), potato (Liu et al., 2014), vanilla (Xiong et al., 2015), and apple (Franke-Whittle et al., 2015) replanting systems. The composition of root exudates varies from plant to plant and affects the relative abundance of microorganisms in association with the roots (Somers et al., 2004). Peanuts, potato, vanilla, and apple are dicotyledonous plant, while the lily is monocotyledonous plants, so this discrepancy may be due to the plant species type.

In this present study, a large number of OTUs could not be identified in L0 and L3 based on the UNITE database which is general used for molecular identification of fungi (Kõljalg et al., 2013). Then we used the NT database to identify these OTUs, and the results also showed the numbers of unidentified OTUs were very high in L0 and L3 (see Supplementary Figure S1). Thus, it is necessary to identify these dark taxa in our future research.

In many crop replant soils, the microorganism population structure shifted with many harmful microorganisms, such as the fungi *Fusarium* spp., *Rhizoctonia solani, Phytophthora* spp., and *Cylindrocarpon* spp. resulting in a negative effect on plant root growth and overall plant health (Mazzola, 1998; Manici et al., 2003; Manici and Caputo, 2010; Chen et al., 2012; Kelderer et al., 2012). The same shift was also observed in this work, which showed that after 6 years of replanting, many identified fungal members, including some important harmful fungal members, such as the genera *Acremonium, Fusarium*, and *Gibberella*, appeared at the expense of a large number of unidentified fungal members that appear to disappeared. This shift resulted in the loss of fungal diversity (Figure 2). Generally, soil biodiversity has a positive correlation with the productivity and sustainability of a system (Hunt and Wall, 2002). For example, a recent report revealed that in comparison to a vanilla monoculture system, a black pepper-vanilla mixed system had significantly higher fungal diversity in terms of both bulk and rhizosphere soils (Xiong et al., 2016). Thus, soil fungal diversity loss and the appearance of some specific fungal communities might be a common phenomenon associated with CRPs.

### Potential Interactions Between Fungal Taxa and Their Replant Environments

Overall, the most dominant phyla *Ascomycota*, *Basidiomycota* and *Zygomycota*, which frequently occur in nature, were also detected in this study (Buée et al., 2009; Li et al., 2009, 2014; Xiong et al., 2015). Among these phyla, *Ascomycota* is found in association with a wide range of crop monoculture systems (Xiong et al., 2016). The class *Sordariomycetes* of *Ascomycota* is most dominant (top 1 class, average RA = 30.90%), which is consistent with many studies that found *Sordariomycetes* to be the most common fungal class in different agricultural systems, and its members are ubiquitous in virtually all ecosystems as pathogens and endophytes of plants (Zhang et al., 2006; Chen et al., 2012; Li et al., 2014).

The phylum *Basidiomycota* includes a vast and complex group of fungi containing a large number of saprophytic (wood decomposers and litter decomposers), ectomycorrhizal, and parasitic fungi. The impact of this phylum on the soil seems complex; for example, it was reported that *Basidiomycota* was correlated with soil properties and vanilla wilt disease (Xiong et al., 2015). In contrast, some reports have found that no correlations are observed between the health status of pea fields and the relative abundance of the phylum (Li et al., 2009; Xu et al., 2012), and in this research, *Basidiomycota* appeared in L6 with high RA (RA = 23.78%), but decreased sharply in L9 (RA = 13.08%); thus, it appears that the impact of this phylum may be specifically related to the particular plant species under cultivation. In this phylum, one major class, *Agaricomycetes* (top 8 class, average RA = 0.60%), which was significantly accumulated in L6 and L9 (RA = 1.16% and 1.23% in L6 and L9, respectively), is reported to be a critical decomposer and contains “soft,” “brown” and “white” rot fungi that produce hydrogen peroxide and enzymes to degrade complex plant compounds, including cellulose and lignin (Sista and Wensheng, 2016). Thus, this class may have caused the increase in lily soil organic matter from the constant organic fertilizer input and residue retention under long-term replanting years. We also detected two other genera, *Cryptococcus* (top 9 genus, average RA = 1.18%) and *Guehomyces* (top 12 genus, average RA = 0.77%) in this phylum, which are oligotrophic organisms and single-celled microorganisms (yeast) and are known for having a wide range of enzymatic activities (Péter and Rosa, 2006; Brandão et al., 2011; Buzzini et al., 2012; Martinez et al., 2016). Yeast have developed adaptation strategies to overcome notably low-nutrient and oxygen-poor conditions, such as those found in oligotrophic lakes in Patagonia and glacial areas (Fonseca and Inácio, 2006). Both of these genera were only detected in L0 with trace RAs and without detection in L3, but they appeared in L6 with high RA and significantly increased in L9 (Figure 5 and Table 1), which might explain why after 6 years of replanting, the soil nutrients degraded severely and these two oligotrophic organisms increased to overcome the low-nutrient conditions. A recent finding demonstrated that the presence of such oligotrophic organisms existed with high RAs in low-nutrient conditions by comparing conventional tillage with reduced tillage (Degrune et al., 2017). Thus, these two genera can be regarded as fungal indicators for soil nutrient degradation in the lily field cropping.

In total, the ecological relevance of the phyla *Ascomycota* and *Basidiomycota* in agroecosystems is understood to some degree, while the ecological relevance of the phylum *Zygomycota* is much less understood. Members of the non-dominant phylum *Chytridiomycota* (RA = 0.36% and 0.20% in L6 and L9, respectively) are commonly found in soil and exhibit either saprobic or parasitic life styles, but its ecological relevance in agroecosystems is still poorly understood (Degrune et al., 2017). A recent study has emphasized the potential ability of the members of this phylum to degrade cellulose, a major component of the plant cell wall, suggesting an important role in organic Carbon decomposition (Sista and Wensheng, 2016).

RDA model revealed that soil properties can strongly influence the fungal communities in the lily continuous cropping soil (Figure 8). All the soil physicochemical properties clearly correlated with the fungal groups identified among them, the salt content is the most important factor impacting on fungal community, and its accumulation is mainly attributed to the increase of available nitrogen and available phosphorus. Soil pH was a key factor that increased with replant years and affected the fungal community composition and structure in this study. This result is similar to the observed phenomenon in vanilla monoculture system (Xiong et al., 2015). Moisture content also structure the fungal communities, which agreed with a recently published study that revealed soil moisture was one important factor explaining bacterium and fungi community structure variations by comparing the microbial communities in conventional tillage with reduced tillage (Degrune et al., 2017).

Organic matter is another important factor in shaping fungal community structure. As we mentioned above, maybe due to the lack of any residue removal and with constant organic fertilizer inputs during perennial cultivation, the organic matter in the lily soil increased over long-term replanting (Table 2). However, total lily fungal abundance and diversity decreased with replant years (Figure 2), which is not supported by some literature, which reported that many fungi use crop residues as a substrate, and organic amendments augment soil organic content and micronutrients, thus directly influencing soil organisms (Titi and Ipach, 1989; Scholte and Lootsma, 1998; Minor et al., 2004). Therefore, the mechanism of the organic matter impact on fungal community structure and function is complex, and the difference may be attributed to crop types and soil types as well as soil and crop residue management practices.

Finally, potassium deficiency is a major contributing factor to limit lily production in the Gansu province region; edible lily requires high available K levels during growth (Lin, 2012). In this study, we show a remarkable decrease in K availability as re-plant continues (Table 2). Since we only identified one fungi genus, *Aspergillus*, associated with potassium assimilation by plants (Velázquez et al., 2016) and it appears to increase in later replant years, we propose that, in lily, fungi may not mediate K availability.

### The Possible Fungal Groups That Cause the Lanzhou Lily CRP

Because each fungal phylum includes a large and complex group of members and each individual might have a different impact on soil, it is difficult to clarify the function of the phyla in replant soil, as some reports have demonstrated (Xu et al., 2012; Li et al., 2009; Franke-Whittle et al., 2015; Xiong et al., 2016). However, many papers have focused on discussing the function of fungal genera related to soil heath in agroecosystems. Here, we similarly explore the possible cause of the lily CRP at the genus level.

The genera *Acremonium, Fusarium, Gibberella, Alternaria, Cryptococcus, Phoma*, and *Ilyonectria* play important roles in the lily CRP. The genus *Acremonium* (top 3 genus, average RA = 5.32%) can cause root rot or replant disease in *Populus*, asparagus, peach and apple (Chakravarty and Hiratsuka, 1994; Blok, 1995; Yang et al., 2012; Trivedi et al., 2016), and the genus *Gibberella* (top 8 genus, average RA = 1.19%) has been reported as a pathogen of apple and peach replants, which includes the perfect stage of several *Fusarium* species (Manici and Caputo, 2010; Tewoldemedhin et al., 2011; Maxin et al., 2012; Manici et al., 2013; Sanzani et al., 2013). The genus *Alternaria* (top 9 genus, average RA = 1.12%) includes many plant pathogens on numerous hosts and its species can cause leaf spots, rots and blights in many crops, such as pomegranate (Ezra et al., 2010), apple (Jung, 2007), pistachio (Ozkilinc et al., 2017), oil palm (Suwannarach et al., 2014), basil (Shinmen et al., 1989), and spoon cabbage (Ko et al., 2010). Another genus, *Phoma* (top 20 genus, average RA = 0.27%), is the largest fungal genus, and plant diseases caused by *Phoma* spp. can occur under favorable environmental conditions and result in leaf and stem spots and significant yield losses (Aveskamp et al., 2008; Li et al., 2013). In addition, *Phoma* spp. have shown higher RAs in fields where peanut was consecutively monocultured (Li et al., 2014). The genus *Ilyonectria* (top 25 genus, average RA = 0.14%) was negatively correlated with plant growth in apple orchards (Chaudhry et al., 2012; Franke-Whittle et al., 2015). In this research, these five genera *Acremonium*, *Gibberella*, *Alternaria*, *Phoma*, and *Ilyonectria* appeared after 6 years of replanting, and their RAs significantly increased in L9; thus, their accumulation might be an important reason for the occurrence of the lily CRP. Moreover, it has been reported that the genera *Cryptococcus* and *Exophiala* (average RA = 0.082%) had high abundances and negative correlations with plant growth in apple replant orchards (Franke-Whittle et al., 2015). As we mentioned above, the presence of the genus *Cryptococcus* is related to the soil low nutrients and oxygen-limited conditions. But as the genus *Exophiala* has low RA, it might not be an important factor in the lily CRP. Where soil-based constraints result in minor pathogens, they cause poor root health but may not lethal, and may be difficult to point to characteristic symptoms, which result in reduced yield in the field (Magarey, 1999). These several genera mentioned here can be described as minor pathogens for lily CRP. A further study will center on their interaction with chemical, physical, and other biological properties.

The result also partially demonstrated that our hypothesis that the accumulation of some *Fusarium* ssp. might play an important role in the lily CRP. *Fusarium* ssp. are well-known fungal pathogens in crop soil-borne diseases. Soils subjected to crop monocultures such as peanut, cucumber and vanilla have accumulated *F. oxysporum*, which results in crops with serious *Fusarium* wilt disease (Zhang et al., 2008; Chen et al., 2012; Xiong et al., 2015). Some *Fusarium* species are reported to result in lily wilt disease (Metson et al., 2008; Yang et al., 2010), including the pathogenic *Fusarium* species *F. oxysporum* and *F. tricinctum* identified in our research (Bian et al., 2016). As the top five genus, *Fusarium* RA was twice as high in L9 (RA = 7.60%) than in L6 (RA = 3.26%). However, further identification at the species level was unexpected; in L6 and L9 we only identified three species, which accounted for 3.26 and 7.60% of the sequences, respectively, and their functions are unknown. Previous studies revealed that high diversity of *Fusarium* spp. is associated with good soil health in permanent tree crops (Macia-Vicente et al., 2008; Kelderer et al., 2012). Therefore, designing specific primer sets to identify specific *Fusarium* species will clarify their role in maintaining the lily soil health, and must be the topic of future research.

Several putative plant-beneficial fungal genera, such as *Tetracladium*, *Penicillium*, *Lecythophora*, and *Paecilomyces*, were detected in this research; *Penicillium* is known to be capable of producing numerous biologically active compounds and to act as a fungal antagonist and plant growth promoter (Khan et al., 2008; Nicoletti and Stefano, 2012; Franke-Whittle et al., 2015). It is also a famous bio-control agent of *Fusarium* wilt disease (Larena et al., 2003; Xiong et al., 2016). In our research, the genus *Penicillium* (top 16 genus, average RA = 0.35%) RA decreased slightly in L9 compared to that in L6. Some reports verified that the genus *Tetracladium* (top 11 genus, average RA = 0.80%) was most likely not pathogenic in terms of replant disease and in fact acted in a beneficial way and increased soil fertility (Franke-Whittle et al., 2015). However, others have reported *Tetracladium as* replant disease soil in apple (Utkhede et al., 1992; Mazzola and Manici, 2012). We therefore can not conclude that, simply on the bases of reduction in RA values (average RA = 2.26% and 0.94% in L6 and L9, respectively), the genus *Tetracladium* is a plant-beneficial fungal group in lily. The genera *Paecilomyces* and *Lecythophora* are positively correlated with plant growth in apple replant orchards (Franke-Whittle et al., 2015), and the genus *Lecythophora* could produce antifungal metabolites that are able to inhibit the growth of pathogenic fungi (Chakravarty and Hiratsuka, 1994). However, we detected these two genera with extremely low RAs (average RA = 0.038%, 0.0002%, respectively), the lack of them, perhaps, is the one to be considered as factors of the lily CRP.

After 6 years of replanting, accumulation of the harbored highly abundant pathogenic fungal genera *Acremonium*, *Fusarium*, *Gibberella*, *Alternaria*, *Cryptococcus*, *Phoma*, and *Ilyonectria* and depletion of the putative plant-beneficial fungal group *Penicillium* is the possible causes of the lily CRP under a 0–9 years replanting system. This result also clearly explains why the lily CRP becomes severe after 6 years of replanting in terms of fungal community degradation. The same result was also observed in similar studies that demonstrated that the accumulation of fungal pathogens occurred at the expense of beneficial plant fungi explained unhealthy crop growth in consecutively monocultured soil (Li et al., 2014; Xiong et al., 2016).

Network analysis revealed that the putative plant-beneficial genus *Penicillium* (degree = 3) co-occurred with all the most keystone genera *Phoma* (pathogenic genus), *Cryptococcus* (oligotrophic and pathogenic genus) and *Monographella* (Figure 7), which indicated the potential interactions among the different fungal taxa. Furthermore, it means that most of these fungal genera were favored in long-term replant system, and these fungal may have shared the same ecological niche or perhaps due to prey–predator interaction.

Finally, in the present study we only discussed several genera that are dominant, and for which their functions in maintaining soil health have been reported. However, in the genera associated with lily’s CRPs, there is still a large amount of unidentified sequences. Thus, based on the distributions and abundances found in this study, further experiments are needed to identify the sequences at species levels and better understand the interaction involving pathogenic and plant-beneficial fungi.

## Conclusion

Lily replanting altered the soil microbial communities: compared to in L0 and L3, the fungal RA and diversity decreased sharply in L6 and L9 at OTUs level. Four to five years of replanting is a key transition period for fungal community structure substantial change that results in shaping completely new fungal community structures in L6 and L9. After 6 years of replanting, a large amount of the unidentified fungal community disappeared, and identified fungal groups appeared at all taxonomic levels, and a loss of fungal diversity and structure simplification occurred too, which are negative to soil health in lily replant production. Moreover, fungal diversity loss and simplification might be common phenomena in crop replant systems. In addition, Soil properties strongly structure the fungal communities in the lily continuous cropping soil and the genera *Cryptococcus* and *Guehomyces* could be regarded as potential indicators to monitor and manage sustainable soil health in this lily cropping system. After 6 years of replanting, accumulation of the harbored highly abundant pathogenic fungal genera and depletion of the putative plant-beneficial fungal groups are the possible causes of the lily CRP. The results suggest that after 3 years of replanting, and especially after 6 years, some specific soil management strategies aimed at encouraging and increasing the beneficial endemic microbial population and preventing harmful fungus accumulation should be implemented efficiently to improve replant soil health. Furthermore, exploring the interaction involving these pathogenic fungal and plant-beneficial fungal members at species levels will give the chance to unravel the causes of the lily CRP.

## Data Availability Statement

The datasets generated for this study can be found in the NCBI SRA under the Bioproject: ID PRJNA550018.

## Author Contributions

GSh and HS designed the research, performed the experiments, analyzed the data, and wrote the manuscript. AC-U, XJ, GSu, and HY participated in discussions and revised the manuscript. All authors contributed to the article and approved the submitted version.

## Conflict of Interest

The authors declare that the research was conducted in the absence of any commercial or financial relationships that could be construed as a potential conflict of interest.

## Footnotes

1 https://unite.ut.ee/

2 https://blast.ncbi.nlm.nih.gov/Blast.cgi

## Supplementary Material

FIGURE S1 Bar-plots showing fungal and bacterial phyla based on NT database: about one half sequences unidentified in L0 and L3, almost all of which belongs to fungal OTUs because there were 99.82% bacterial sequences identified in the same soil samples (unpublished data).
