# Supplementary figures and images for: Soil Fungal Diversity Loss and Appearance of Specific Fungal Pathogenic Communities Associated With the Consecutive Replant Problem (CRP) in Lily
Source: Front Microbiol. 2020 Jul 15;11:1649. doi: 10.3389/fmicb.2020.01649 (PMC7373732; doi:10.3389/fmicb.2020.01649)

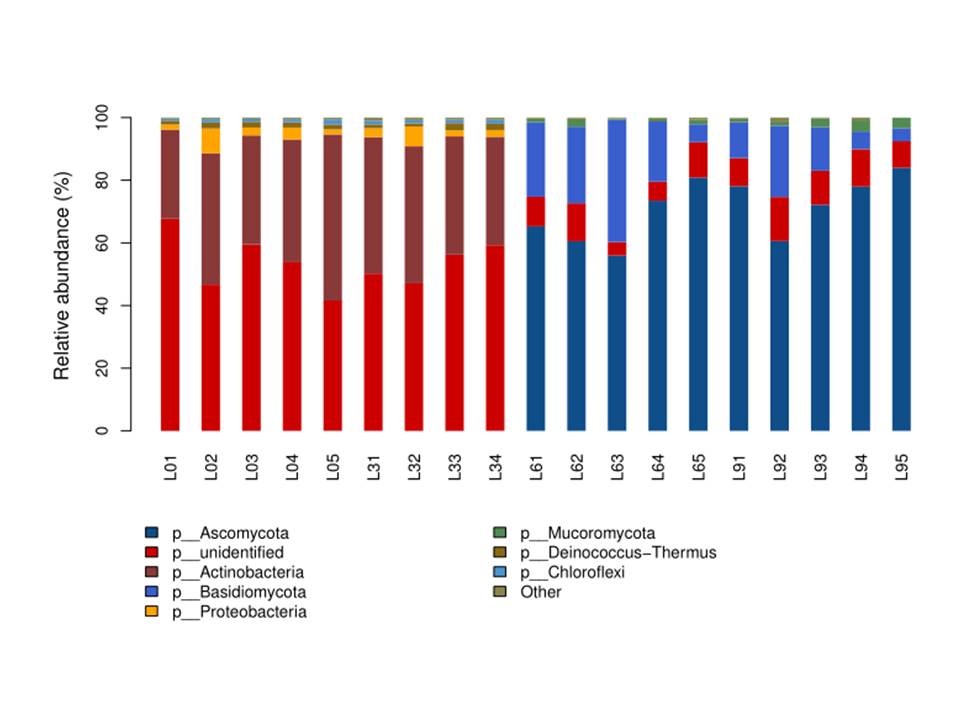

Supplement: FIGURE S1 — Bar-plots showing fungal and bacterial phyla based on NT database: about one half sequences unidentified in L0 and L3, almost all of which belongs to fungal OTUs because there were 99.82% bacterial sequences identified in the same soil samples (unpublished data). [file Image_1.JPEG]
